# Association of Plasma Heat Shock Protein 70, Interleukin 6, and Creatine Kinase Concentrations in a Healthy, Young Adult Population

**DOI:** 10.1155/2015/967120

**Published:** 2015-11-18

**Authors:** Carmen Contreras-Sesvold, Bradley D. Revenis, Francis G. O'Connor, Patricia A. Deuster

**Affiliations:** Human Performance Laboratory, Department of Military and Emergency Medicine, Uniformed Services University of the Health Sciences, 4301 Jones Bridge Road, Bethesda, MD 20814, USA

## Abstract

Variations of baseline plasma concentrations of creatine kinase (CK), heat shock protein 70 (HSP70), and interleukin 6 (IL-6) have been reported. We report categorical associations which may influence these protein levels.* Methods*. Blood was harvested for DNA and plasma protein analysis from 567 adults. Mean protein levels of CK, HSP70, and IL-6 were compared by sex, ethnicity, genetic variants—CKMM Nco1 (rs1803285), HSPA1B +A1538G (rs1061581), and* IL6* G-174C (rs1800795)—self-reported history of exercise, oral contraceptive use, and dietary supplement use.* Results*. SNP major allele frequencies for CKMM, HSPA1B, and* IL6* were 70% A, 57% A, and 60%. Mean CK statistically differed by sex, ethnicity, oral contraceptives, and caffeine. Plasma HSP70 differed by caffeine and protein. Mean IL-6 concentration differed by sex, ethnicity, and genotype. Plasma IL-6 was significantly lower (29%) in males (1.92 ± 0.08 pg/mL) and higher (29%) among African Americans (2.85 ± 0.50 pg/mL) relative to the others.* IL6* G-174C GG genotype (2.23 ± 0.14 pg/mL) was 19% greater than CG or CC genotypes.* Conclusion*. Differences in baseline CK and IL-6 plasma protein concentrations are associated with genetics, sex, ethnicity, and the use of oral contraceptives, caffeine, and protein supplements in this young and athletic population.

## 1. Introduction

Exertional Rhabdomyolysis (ER) affects 1 in 10,000 people per year [1] and is a significant threat for military personnel and civilian fire and medical service responders during training, particularly when the training is under warm-to-hot environmental conditions [2–4]. Importantly, the incidence of ER in the military population has more than tripled between 2006 and 2011 [4]; thus, it is a concern for military readiness. Further, exertion under various environmental and physical conditions induces multiple key proteins as either indicators of stress, signals for metabolic pathways, or protective mechanisms. Proteins known to be increased by exertional stress include creatine kinase, heat shock protein 70 (HSP70), and interleukin 6 (IL-6) [5–10]. High plasma creatine kinase (CK) activity levels are considered a clinical marker of exercise-induced muscle damage [11] with some individuals demonstrating extreme elevations following strenuous exercise [5, 12]. Likewise, baseline CK levels may have significant variability ranging from 50 to 1,200 U/L [13]. Heat shock protein 70 (HSP), a highly conserved family of stress-responsive proteins, is induced by both exercise and heat exposure [8, 10, 14, 15]. Interleukin 6 (IL-6), a cytokine and stress-responsive myokine, is released from contracting skeletal muscles, perhaps acting as an energy sensor, to exert both local and endocrine metabolic effects [16]. Both baseline and exercise-induced levels of these proteins can be influenced by multiple factors including diet, exercise fitness, overall health status, sex, medications, ethnicity, and genetics [17].

Importantly, baseline protein concentrations may be the key to understanding how such proteins change in response to environmental exposures and extreme physical exercise. Studies have shown that baseline CK levels differ by sex and ethnicity [18] and that both baseline [19] and exercise-induced CK [5, 12] have genetic determinants. Variations in constitutive and inducible HSP70 levels have been demonstrated among individuals of different athletic abilities [8, 10] and appear to be upregulated in response to heat acclimation [15]. Likewise, many factors affect baseline and stress-induced levels of IL-6, including hypothalamic pituitary adrenal axis function, immune status, and possibly diet [9, 20]. Thus, identifying confounding factors that affect baseline levels of key stress proteins is important for understanding subsequent responses to single and multiple stressors.

The genes that code for these three important stress-associated proteins have well-described polymorphisms that have been examined with regard to various phenotypes. The muscle-specific creatine kinase gene (*CKMM*) has 3′ A to G single nucleotide polymorphism (SNP), which has been studied with regard to performance and muscle damage [21, 22]. This 3′ sequence has been associated with mRNA localization of the CK protein [23], and differential localization of CK could potentially alter metabolic balance. One HSP70 gene,* HSPA1B*, has a synonymous variant, +A1538G (or +A1267G), which has been studied with regard to heat tolerance, high-altitude pulmonary edema, and clinical outcomes after severe injury [24–26]. The gene that codes for IL-6,* IL6*, has a well-described SNP located within its promoter, G-174C, which may determine baseline and/or IL-6 levels in response to stress [27–30].

In an effort to identify biomarkers for ER, base line values in a healthy athletic population must first be characterized. The objective of this study was to determine the contribution of genetic variation to baseline plasma concentrations of CK, HSP70, and IL-6. We also examined differences in protein levels as a function of sex, ethnicity, oral contraceptives, exercise regimen, supplements, and genetics in a group of Marine Corps Officer Candidates.

## 2. Methods

### 2.1. Participants

Participants were apparently healthy, physically active students in Marine Corps Officer Candidate School, aged 18–33 years (*n* = 567). Informed consent was obtained from all participants prior to the beginning of the study. The study was approved by the Institutional Review Boards of the National Naval Medical Center and Uniformed Services University of the Health Sciences (USUHS), Bethesda, MD, USA.

### 2.2. Questionnaires

Participants filled out one-page questionnaire covering demographics (age, sex, ethnicity, height, and weight), along with relevant questions about current health status and health behaviors. These questions included items about sleep, recent illnesses, medication (including use of oral contraception), exercise history, exercise regimen, exercise related injuries (including EHI and rhabdomyolysis), supplementation (caffeine or nutritional supplements), menstrual status/cycle questions (women), and SCT.

### 2.3. Blood Handling and Processing

Blood was drawn in the morning on the first day of medical in-processing and before any physical training for the candidates. It was collected in EDTA-containing tubes and stored on ice for transport (~1 hour). Upon arrival, it was immediately centrifuged for buffy coat isolation. Plasma was aliquoted for protein analysis and stored at −80°C until being ready for use. Buffy coats were transferred to 1.5 mL centrifuge tubes and stored (<48 hours) at 4°C until further processing.

DNA was extracted from the buffy coat using the QIAamp DNA mini kit 250 as per package protocol (Catalog # 51106 Qiagen, Valencia, CA, USA). DNA was harvested from 455 samples. DNA concentrations were measured via spectrophotometry at 260 nm (NanoDrop ND-1000, ThermoFisher Scientific, Wilmington, DE). DNA purity was assessed by 260/280 nm; ratios within 1.80 to 1.85 were considered acceptable. Samples out of tolerance were reextracted as described above. Portions of DNA stock solutions were subsequently diluted to 25 ng/*μ*L to create standardized working stock solutions.

### 2.4. Genotyping

Analysis of single nucleotide polymorphisms (SNP) for* CKMM* Nco1 A^*∗*^ to G (rs1803285) [5] *n* = 453,* HSPA1B* +A^*∗*^1538G (MP1 assay) [31] (rs1061581) *n* = 455, and* IL6 *G^*∗*^-174C (rs1800795) [30] *n* = 454 was accomplished as previously described [5, 30, 31]. Asterisks denote the major alleles. Amplicons could not be generated from all samples presumably because polymorphisms existed within the primer locations or DNA was no longer available.

### 2.5. Protein Analyses

Plasma CK protein activity levels were measured as previously described [5]. Plasma HSP70 concentrations were assayed by using the HSP70 High Sensitivity EIA kit (Catalog # EKS-715, ENZO life Science, Ann Arbor, MI, USA) as per package protocol. Detection range of the HSP70 assay was 0.20–12.50 ng/mL; the intra- and interassay coefficients of variation were <10 and <6%, respectively. Plasma IL-6 protein (IL-6) concentrations were assayed using the Quantikine HS Human IL-6 kit (Catalog # SS600B, R&D Systems, Minneapolis, MN, USA) as per package protocol. Detection range of the IL-6 assay was 0.156 to 10 pg/mL; the intra- and interassay coefficients of variation were <10 and <7%, respectively. Any sample above the detection limit for the HSP70 or IL-6 assay was rediluted to be within the detection limit and reassayed as per assay instructions. All HSP70 and IL-6 assays had the same duplicate control sample to validate interassay results.

### 2.6. Statistical Analyses

The Hardy-Weinberg Equilibrium equation was used to verify SNP normal genotypic distribution. Fisher's Exact Test and *χ* ^2^ were used to compare the allele distribution with other published studies. The categorical influence on protein concentrations was performed by ANOVA and/or independent samples *t*-test when applicable. BMI was a covariate for all protein analyses. Protein values generally exhibit a nonnormal distribution and thus all data were log-transformed for analysis; however data were reported as mean ± standard error (SE). All statistical tests were performed using SPSS software V.20 (SPSS, Inc., Chicago, IL, USA). Samples defined as outliers (values ≥ ±3 standard deviations) were removed from analyses; this included 6 CK, 8 HSP70, and 4 IL-6 with cutoff values of 520 U/L, 48 ng/mL, and 7.40 pg/mL, respectively. No two outliers belonged to any one participant. Body mass index (BMI) was calculated as weight in kg/(height in m)^2^. All statistical analyses were verified by USUHS Biostatics Consulting Center; statistical power was 80% or greater for all analyses.

## 3. Results

### 3.1. Population Characteristics

Table 1 describes the general characteristics of the Marine Officer cohort. The sample population (*n* = 567) was predominately male (78.5%) relative to females (10.1%); 65 participants did not report their sex. Overall, 76% were Caucasian (CA) (*n* = 433), 8% Hispanics (HI) (*n* = 44), 6% Asians (AS) (*n* = 32), 4% African Americans (AA) (*n* = 20), 3% Mixed Race/Other (*n* = 18), 2.8% nonreporting (*n* = 16), and <1% Native Americans (*n* = 4). Ethnic samples identified as Native American, Mixed Race, Other, and those nonreporting were excluded from ethnic analyses because of the small sample size. BMI was significantly different by sex (*F*(1,482) = 20.6, *p* < 0.00) and revealed a trend by ethnicity (*F*(3,462) = 2.3, *p* = 0.07) with values for AA, AS, CA, and HI being 25.4, 23.6, 24.4, and 25.0 kg/m^2^, respectively. BMI also demonstrated a significant difference (*p* < 0.05) by* IL6* genotype CC: 24.2, CG: 24.0, and GG: 25.0.* HSPA1B* and* CKMM* were not significantly different by genotype.

### 3.2. Ethnicity

Table 2 presents the plasma protein concentration values by ethnicity. As expected, CK was significantly higher in AA (*F*(3,484) = 8.79, *p* < 0.00). Plasma IL-6 was also significantly different with regard to ethnicity (*F*(3,236) = 4.39, *p* < 0.00) with CA having the lowest and AA having the highest concentrations. In contrast, HSP70 (*p* > 0.05) did not differ significantly by ethnicity. However, HSP70 values for AA were approximately 33% lower relative to other ethnic groups. The influence of BMI on CK, IL-6, and HSP70 concentration values was not significant (*p* > 0.05).

### 3.3. Sex

Significant sex differences were noted for both CK and IL-6. Mean CK concentrations were significantly higher (*F*(1,463) = 9.1, *p* < 0.00) for males (144 ± 4 U/L) than for females (106 ± 10 U/L). In women, the use of oral contraceptives was associated with a significant increase (39%) in CK (125 ± 17 versus 90 ± 13 U/L) (*F*(1,49) = 4.5, *p* < 0.05) as compared to those that reportedly did not use them. There was no significant difference in CK concentrations between females that used oral contraceptives and males (*t*(434) = 0.689, *p* > 0.05). Mean IL-6 concentrations were significantly lower (*F*(1,237) = 11.8, *p* < 0.01) in males (1.92 ± 0.08 pg/mL) as compared to females (2.69 ± 0.23 pg/mL). Mean HSP70 did not differ significantly (*p* > 0.05) by sex (male 6.6 ± 0.5 versus female 6.0 ± 0.5 ng/mL). BMI did not significantly (*p* > 0.05) alter these results.

### 3.4. Genotype

**Figure 1:**
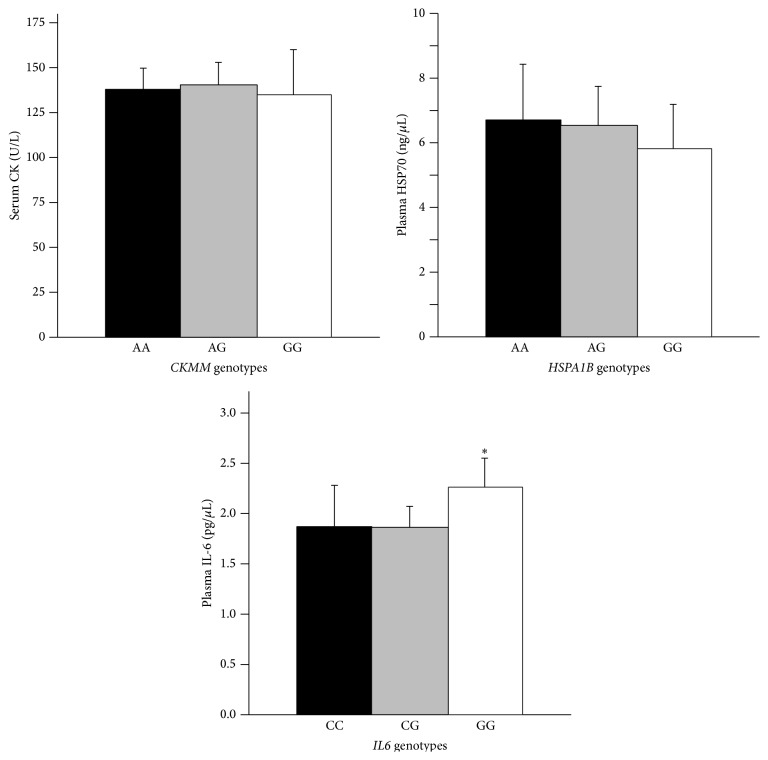
These figures represent the mean (+SE) plasma creatine kinase (CK) activity (U/L) by* CKMM* genotype, HSP70 by* HSPA1B *genotype, and IL-6 by* IL6* genotype. CK and HSP70 did not differ by genotype (ANOVA). IL-6 GG genotype was significantly different (*p* < 0.05) than the combined CC and CG genotype (*t*-test).

Distributions of the genotypes for the* CKMM*,* HSPA1B*, and* IL6* polymorphism were all in Hardy-Weinberg Equilibrium (HWE) conditions, with *χ* ^2^ values of 0.28, *p* = 0.59; 0.003, *p* = 0.96; and 1.79, *p* = 0.18, respectively. All ethnic specific genotype frequencies were also in HWE (*p* > 0.05).  Table 3 shows the genotypic distributions of the targeted polymorphisms by ethnicity. The genotypic distributions for the* HSPA1B* and* IL6* genes differed such that the percentages of GG genotypes for both SNPs were significantly higher in AA than AS, CA, and HI (*HSPA1B*: 56% versus 14%, 17%, and 27%;* IL6*: 80% versus 62%, 32%, and 46%, resp.).  Figure 1 presents protein concentrations by their respective genotypes. No significant differences in protein levels were noted for* CKMM* or* HSPA1B p* > 0.05 by genotype. However,* IL6* displayed a trend by genotype (*F*(2,249) = 3.027, *p* = 0.05), and mean plasma IL-6 levels were significantly lower for C+ genotypes (1.87 ± 0.09 pg/mL) than the GG genotype (2.26 ± 0.14 pg/mL) (*t*(250) = −2.5, *p* < 0.05). BMI did not significantly alter these results (*p* > 0.05).

### 3.5. Supplements and Exercise History

Those participants that answered yes (*n* = 49) to the question “Do you Drink more than three caffeinated beverages per day?” revealed a trend for less CK (119 ± 12 versus 140 ± 4 U/L) as compared to those that reported no (*t*(505) = −1.9, *p* = 0.06). They also had significantly reduced HSP70 (4.5 ± 0.7 versus 6.6 ± 0.6 ng/mL) (*t*(186) = −2.1, *p* < 0.05). IL-6 was not significantly different for this subset (*p* > 0.10). Those that reported protein supplementation (*n* = 18) for this population had significantly higher HSP70 (14.6 ± 8.4 versus 6.3 ± 0.4 ng/mL) (*t*(189) = 2.0, *p* < 0.05) than others. No significant difference was found for IL-6 and CK. Those that reported the use of vitamins (*n* = 28) or creatine supplementation (*n* = 10) also did not have significantly different CK, HSP70, or IL-6 levels than the others. No significant differences of CK, HSP, and IL-6 protein levels were found for those who reported performing aerobic exercise (95%) only or strength training (92%) only or both aerobic exercise and strength training together as compared to those nonreporting (*p* > 0.05). No protein differences (*p* > 0.05) were found for those (>30%) that reported “exercising strenuously with in [*sic*] the past 2-3 days”.

## 4. Discussion

We initially sought to determine whether baseline concentrations of three proteins found in plasma with important functions relative to exertional stress differed by sex, ethnicity, and well-characterized SNPs in a population of healthy, young adults. We further explored the influence of exercise history, oral contraceptives, and dietary supplementation for this cohort on these proteins.

As expected, plasma CK concentration differed significantly by sex and ethnicity, but not by the* CKMM* genotype. Females represent 11% of this study's population and their mean CK levels were significantly lower than the male population (~20%), which is in agreement with other studies [32, 33]. However, if we added the influence of oral contraceptives, females who used oral contraceptives had similar CK values to males. While these values are consistent with the literature [34, 35], this is an area which requires more investigation. Likewise, our ethnic differences are similar to those reported by others [18, 36–38], with AAs having the highest levels followed by AS, CA, and HI. Our mean concentrations are higher than others have reported, but our population was both young and beginning military training, as compared to older populations in other studies [18, 33]. Interestingly, our mean CK value for HI was higher (129 ± 16 U/L versus 41.5 ± 36 U/L) than another published study [39], but our study's average age for HI was ~22 years as compared to ~47 years in their study [39]. CK levels decline with age [18] so the difference is understandable. Further mean differences between studies may reflect the physical activity [40] of this population, as >92% of our participants reportedly exercise at least twice per week. Baseline CK levels did not differ by genotype as previously reported [5]. Caffeine use was associated with a trend for reduced CK levels. We are currently exploring the use of caffeine with exercise and heat in a larger sample size to dissect the physiological effects.

No significant differences for HSP70 concentrations were noted across sex, ethnicity, or genotype with our mean HSP70 protein levels in agreement with previous studies [41]. To our knowledge, variations in HSP70 by ethnicity have not been previously been reported. Further, the genetic distribution for* HSPA1B* is in agreement with previous work [31], but AA had a significantly greater percentage who were homozygous for the minor allele. Interestingly, a significant difference for reduced HSP70 concentrations was associated with caffeine use. Again we are currently exploring this phenomenon for a more complete picture of caffeine's physiological effects. We also present a new finding that protein supplementation was associated with a significant increase in HSP70 and no other covariable was associated or could explain this phenomenon. Increases in HSP70 are associated with variable health outcomes. On the one hand, elevated HSP70 concentrations have been associated with increased longevity, positive postsurgical outcomes, and cardiac and brain protection from ischemia [42]. On the other, increases have been associated with inflammation, preeclampsia, and enhanced cancer progression [43–45]. Again this phenomenon will require further investigation.

Plasma IL-6 levels differed by ethnicity, sex, and genotype, with AA and those with the GG genotype having the highest values relative to other ethnicities and the C+ genotypes, respectively. Our results were not altered as expected after adjusting for BMI (*p* > 0.05). Our mean IL-6 concentrations overall and by sex are consistent with other control populations [29, 46] as were values for CA and AA [29, 47]. Further, our mean IL-6 concentrations for AS are higher than previously reported by Ognjanovic et al. [48] and higher for all ethnicities when compared to those reported by Wong et al. [49], but these differences may reflect participant differences in age, physical fitness, and other characteristics. Importantly, persons homozygous for the G allele had significantly higher IL-6 levels than those with the C allele. This finding is consistent with two other studies [28, 50] and somewhat consistent with one other report that found that persons with the CC genotype had significantly lower IL-6 levels than those with the GG or CG genotypes [27]. However, it is in contradiction to the finding of Ognjanovic et al. [48] who found no significant differences. Clearly multiple factors affect baseline values: inflammation, menstrual status, obesity, physical activity, and age [46, 48, 51]. It is important to note that participants in this study were young, normal-weight, healthy, and physically fit, so the baseline proteins may not be comparable to other studies examining older more sedentary populations. Further research will be required to address this disparity.

It is well established that exercise alters CK, HSP70, and IL-6 protein levels [5, 8, 10, 12, 14, 16]. However, no protein differences were found in this population in response to reported baseline exercise regimens or recent strenuous exercise. This may be in part because those that completed this portion of the questionnaire (97.5%) reported they exercised regularly, while the remainder (2.5%) left this section blank. Further, approximately 93% of the cohort reported performing aerobic exercise in combination with strength training regularly; thus this population is fairly homogenous with regard to exercise regimen so it is not surprising that the protein levels reflect this. Moreover, sample size was not sufficient to detect differences in protein levels for those that reportedly performed aerobic exercise only (7%) or strength training only (0.5%).

Limitations to the study include that we did not control for age, mental status, diet, and menstrual status. We looked exclusively at genotype, sex, ethnicity, oral contraceptive use, dietary supplementation (caffeine, protein, and creatine), and plasma protein concentrations. In addition, while the population had yet to begin the rigors of officer candidate basic training, we were unable to control activity levels in the previous days prior to medical in-processing.

## 5. Conclusion

Concentrations of plasma proteins often associated with ER are affected by a variety of factors, including sex, ethnicity, genetic variants, diet, exercise, and medications. This study documents the baseline protein concentrations of CK, HSP70, and IL-6 and the effect of sex, genotype, and ethnicity as well as exercise, oral contraceptives, and dietary supplements on these concentrations from a young, healthy, physically fit, and diverse adult population. In a cohort of young male and female Marine Corps Officer Candidates, the major allele frequencies of polymorphisms for* CKMM*,* HSPA1B*, and* IL6* were 70% A, 57% A, and 60% G, respectively. Mean CK levels differed by sex and ethnicity, but not genotype. African Americans and Asians had 57% and 30% higher CK levels, respectively, than the study mean. Females that used oral contraceptives have CK values similar to their male counterparts in this population. Regular caffeine consumption coincided with reduced plasma CK and HSP70 values, while reported dietary protein supplementation increased HSP70 levels in the blood. However, plasma HSP70 did not differ by genotype, sex, or ethnicity. Mean IL-6 concentration differed by sex, ethnicity, and genotype. Plasma IL-6 was 30% higher in females and almost 40% higher among African Americans as compared to the study mean.

This report establishes normal protein concentration ranges which can be used as reference guidelines for future ER studies on the effect of heat, exercise, nutraceuticals, and other physiological modulators.

## Figures and Tables

**Table 1:** Population characteristics.

General characteristics	Males	Females	All
Age (yrs.)	21	22	21
Weight (kg)	78.2 ± 0.4	62.3 ± 0.8	76.5 ± 0.5
Height (cm)	178.6 ± 0.4	165.1 ± 0.9	177.0 ± 0.4
BMI	24.55 ± 0.13	22.88 ± 0.29	24.4 ± 0.13

**Table 2:** Plasma protein concentrations (mean ± SE) of creatine kinase, heat shock protein 70, and interleukin-6 by ethnicity.

Ethnicity	Creatine kinase (U/L)	Heat shock protein 70 (ng/mL)	Interleukin 6 (pg/mL)
African American (19)	218 ± 28	4.2 ± 0.6	2.85 ± 0.50
Asian (31)	179 ± 17	7.7 ± 1.8	2.07 ± 0.18
Caucasian (395)	131 ± 04	6.3 ± 0.4	1.90 ± 0.09
Hispanic (43)	129 ± 15	6.6 ± 2.0	2.53 ± 0.26
All (488)	138 ± 04	6.4 ± 0.4	2.06 ± 0.08

**Table 3:** Genotypic distribution by ethnicity.

Ethnicity	*CKMM*			*HSPA1B*			*IL6*		
	AA	AG	GG	AA	AG	GG	GG	CG	CC
African American	18.8% (3)	56.3% (9)	25.0% (4)	0% (0)	43.8% (7)	56.3% (9)	80.0% (12)	13.3% (2)	6.7% (1)
Asian	65.5% (19)	31.0% (9)	3.4% (1)	24.1% (7)	62.1% (18)	13.8% (4)	62.1% (18)	37.9% (11)	0% (0)
Caucasian	50.4% (174)	40.6% (140)	9.0% (31)	35.0% (121)	48.6% (168)	16.5% (57)	31.8% (110)	47.4% (164)	20.8% (72)
Hispanic	43.8% (14)	46.9% (15)	9.4% (3)	30.3% (10)	42.4% (14)	27.3% (9)	45.5% (15)	45.5% (15)	9.1% (3)

All	49.8% (210)	41.0% (173)	9.2% (39)	32.5% (138)	48.8% (207)	18.6% (79)	36.6% (155)	45.4% (192)	18.0% (76)

This table represents the % distribution of genotypes for each ethnicity. Parentheses indicate sample size. Major alleles are as follows: *CKMM*: A; *HSPA1B*: A; *IL6*: G.
